# Dataset of two experiments of the application of gamified peer assessment model into online learning environment MeuTutor

**DOI:** 10.1016/j.dib.2017.04.032

**Published:** 2017-04-29

**Authors:** Thyago Tenório, Ig Ibert Bittencourt, Seiji Isotani, Alan Pedro, Patrícia Ospina, Daniel Tenório

**Affiliations:** a Federal University of Alagoas (UFAL), Campus Arapiraca/Pólo Penedo, Av. Beira Rio, Centro, 57200-000 Penedo, AL, Brazil; b Computing Institute, Federal University of Alagoas (UFAL), Campus A.C. Simões, Cidade Universitária, 57072-970 Maceió, AL, Brazil; c Institute of Mathematics and Computer Science, University of São Paulo (USP), Avenida Trabalhador São Carlense, 400, Centro, 13566-590 São Carlos, SP, Brazil; d Center of Exact and Natural Sciences, Federal University of Pernambuco, Av. Prof. Moraes Rego, 1235, Cidade Universitária, 50670-901, Recife, PE, Brazil

**Keywords:** Gamified peer assessment model, Gamification, On-line learning environments, Modelling, Statistical experiments

## Abstract

In this dataset, we present the collected data of two experiments with the application of the gamified peer assessment model into online learning environment MeuTutor to allow the comparison of the obtained results with others proposed models. MeuTutor is an intelligent tutoring system aims to monitor the learning of the students in a personalized way, ensuring quality education and improving the performance of its members (Tenório et al., 2016) [1]. The first experiment evaluated the effectiveness of the peer assessment model through metrics as final grade (result), time to correct the activities and associated costs. The second experiment evaluated the gamification influence into peer assessment model, analyzing metrics as access number (logins), number of performed activities and number of performed corrections. In this article, we present in table form for each metric: the raw data of each treatment; the summarized data; the application results of the normality test Shapiro–Wilk; the application results of the statistical tests *T*-Test and/or Wilcoxon. The presented data in this article are related to the article entitled “A gamified peer assessment model for on-line learning environments in a competitive context” (Tenório et al., 2016) [1].

**Specifications Table**

**Table:** 

Subject area	Computer Science
More specific subject area	Methodology and Technical Computing
Type of data	Tables
How data was acquired	Data were automatically collected within online learning environment MeuTutor
Data format	Raw and analyzed
Experimental factors	Data of six different metrics (final grade, time to correct the activities, associated cost, access number, number of performed activities and number of performed corrections) from two experiments of the application of the gamified peer assessment model into online learning environment MeuTutor were analyzed using the R tool for statistical analysis.
Experimental features	The raw data obtained from execution of the experiments were analyzed using the R tool. At first moment, the data were summarized for a behavior׳s descriptive analysis, where box diagrams were generated. Then, we analyzed the behavior (normality) of the data using the Shapiro–Wilk test [2]. Finally, we used the statistical tests *T*-Test [3] and Wilcoxon [4] to find the *p*-value and to analyze the data from statistical form.
Data source location	Brazil
Data accessibility	The data are available with this article

**Value of the data**

• The data shows that the average grade given by students to an activity is equivalent to the grades given by experts. Moreover, the time and associated costs to complete an evaluation were largely reduced. In this sense, the data can be reused for comparison purposes with the application of another proposed peer assessment model.
• The use of gamification increased the number of students׳ access, the number of performed/submitted activities and the quantity and quality of the evaluations for each activity. These data could be used for developing improved strategies to maximize gains of gamification by varying the used elements.
• The presented data in this article can be used in a comparative way (as a benchmark), comparing with results of future applications of the proposed gamified peer assessment model in others online learning environments and/or different application contexts.

## 1 Experimental design, materials and methods

The proposed gamified peer assessment model was implemented and integrated with the online learning environment MeuTutor, which is an intelligent tutoring system aims to monitor the learning of the students in a personalized way, ensuring quality education and improving the performance of its members [1]. The model was applied in the correction of activities of the students involved in the online learning environment MeuTutor.

Gamification ensures students to feel motivated to participate performing activities and correcting them. We defined the following targets behaviors: To encourage students to participate more often of the activities; To challenge students to get badges; To provide medals to students who participate of the activities; To provide badges or points to students for answer/correct activities; To promote a ranking parametrized for students; To encourage competition for students, with rewards to the winners; The used gamification elements were points, badges, missions, rankings and medals. All information of how to get them are detailed in the research article [1].

Two experiments were conducted. At first, thirty students participated of the process. The submitted activity correction process by students was varied composed by three treatments: T1 (correction by the teacher within the online learning environment MeuTutor); T2 (correction using the proposed gamified peer assessment model within the online learning environment MeuTutor); T3 (manually correction by teacher without the online learning environment MeuTutor). The analyzed metrics in this experiment were the value of the final grade, the time spent for activity correction and the cost involved in the process. The data of the analyzed metrics were obtained from environment MeuTutor and, in the case of treatment T3, through a Google Docs form.

The second experiment aimed to evaluate the gamification within the proposed peer assessment model. This experiment was performed with high school students. After configuration of the environment MeuTutor, a training was performed where the environment was presented to directors, coordinators, teachers and all the students of the high school classes (separately). The nearly one hundred students who were present were interested to participate. Randomly and for each class, we distributed one group to each student (1 or 2), representing the control group (CG) and test group (TG).

The control group students performed the treatment T1 (without gamification), while test group students performed the treatment T2 (with gamification). It is noteworthy that both used the proposed peer assessment model and the only difference is on the presence or not of gamification elements. The analyzed metrics in this experiment were the number of access (logins), number of performed activities and number of corrected activities. The data from this experiment were also directly obtained from the educational environment MeuTutor.

The obtained raw data from experiments were analyzed using the R tool. At first moment, the data were summarized for a behavior׳s descriptive analysis, where box diagrams were generated. Then, we analyzed the behavior (normality) of the data using the Shapiro–Wilk test [2]. Finally, we used the statistical tests *T*-Test [3] and Wilcoxon [4] to find the *p*-value and to analyze the data from statistical form. A *p*-value of 0.05 was used for all statistical analyzes.

Publish the experiments data allows new researchers to compare the results of applying their peer assessment models with the proposed model results in this work [1]. In addition, it is possible in future researches to adapt the proposed model, especially with respect to the gamification elements, obtaining new results. Thus, the presented results in this paper serves as a basis for the evaluation of future models and adaptation of this proposed model.

For the reuse of such data the authors can obtain the results of similar metrics in their researches and compare with the presented results in this paper. It is possible, for example, to know if the other proposed model takes less time and is less expensive than the presented model. It is also possible to compare results between similar models, varying only the gamification. For example, is the researcher X model that uses points and trophies more motivating than the researcher Y model that uses rankings and missions?

## 2 Data files organization

Two XLSX files are presented. The files are separated according to the experiment which it refers. The first one refers to the first experiment and filename is “Experiment_one”. Similarly, the second one refers to the second experiment and the filename is “Experiment_two”.

The experiment 1 had the metrics: final grade (activity final grade given by the proposed model and/or teacher); involved time (time to correct one performed activity); associated cost (cost to correct one activity). The metrics were analyzed in relation to three treatments: T1 (correction by the teacher within the online learning environment MeuTutor); T2 (correction using the proposed gamified peer assessment model within the online learning environment MeuTutor); T3 (manually correction by teacher without the online learning environment MeuTutor). The final grade (grade) metric means the final grade that the student achieved for their activity. It ranges from 0 to 1000. The involved time (time) metric means the time in seconds that an activity took to be responded. Finally, the associated cost (cost) metric means the cost in Brazilian Reais (R$) spent with the teachers (when applied) and/or in the system to correct an activity of the students.

On the other hand, the experiment two had the metrics: access number (number of times the user entered the system); number of performed activity (number of activities a user performed); number of performed corrections (number of activities a user corrected). The metrics were analyzed in relation to two treatments: students in the control group (CG) performed the treatment T1 (without gamification), while students in the test group (TG) performed the treatment T2 (with gamification). The amount of access metric (access) means the number of accesses that the student had in the system (start with 0 without final limit). The performed activity metric (performed activity) means the number of activities the student has made in the system, ranging from 0 to 3. Finally, the corrections performed metric (performed corrections) means the number of activities that the student corrected in the system, (start with 0, limited to number of activities made by students).

In each experiment file, the metrics are indicated by Tabs. For example, the file “Experiment_one” has the Tabs “Metric grade”, “Metric Time” and “Metric cost”, representing each metric cited above. The data of the three treatments of experiment one (T1, T2 and T3) with respect to each metric are presented in respective Tabs. The paper presents for each metric: The raw data of each treatment; The summarized data; The results of the application of Shapiro–Wilk normality test; The results of the application of statistical tests *T*-Test and/or Wilcoxon; In case of Experiment Two, there is still one tab that presents some data of a descriptive manner. A complete description of the data and methods is presented elsewhere [1].

The files mean the raw data measured with the application of the experiments in relation to the evaluated metrics. They may serve as a benchmark for other researches to evaluate the effectiveness of their gamification methods, for example. The other CSV files refer to obtained data after statistical analysis.
